# Efficacy of brief alcohol screening intervention for college students (BASICS): a meta-analysis of randomized controlled trials

**DOI:** 10.1186/1747-597X-7-40

**Published:** 2012-09-12

**Authors:** Alexandre Fachini, Poliana P Aliane, Edson Z Martinez, Erikson F Furtado

**Affiliations:** 1 Department of Neurosciences and Behavior, University of Sao Paulo, Av. dos Bandeirantes, 3900 – 3° andar, Monte Alegre, Ribeirão Preto, SP 14048-900, Brazil; 2 Department of Social Medicine, University of Sao Paulo, Av. dos Bandeirantes, 3900 – 2° andar, Monte Alegre, Ribeirão Preto, SP, 14048-900, Brazil

**Keywords:** Alcohol, Brief intervention, BASICS, College, Prevention, Meta-analysis

## Abstract

**Background:**

Many studies reported that brief interventions are effective in reducing excessive drinking. This study aimed to assess the efficacy of a protocol of brief intervention for college students (BASICS), delivered face-to-face, to reduce risky alcohol consumption and negative consequences.

**Methods:**

A systematic review with meta-analysis was performed by searching for randomized controlled trials (RCTs) in Medline, PsycInfo, Web of Science and Cochrane Library databases. A quality assessment of RCTs was made by using a validated scale. Combined mean effect sizes, using meta-analysis random-effects models, were calculated.

**Results:**

18 studies were included in the review. The sample sizes ranged from 54 to 1275 (median = 212). All studies presented a good evaluation of methodological quality and four were found to have excellent quality. After approximately 12 months of follow-up, students receiving BASICS showed a significant reduction in alcohol consumption (difference between means = −1.50 drinks per week, 95% CI: -3.24 to −0.29) and alcohol-related problems (difference between means = −0.87, 95% CI: -1.58 to −0.20) compared to controls.

**Conclusions:**

Overall, BASICS lowered both alcohol consumption and negative consequences in college students. Gender and peer factors seem to play an important role as moderators of behavior change in college drinking. Characteristics of BASICS procedure have been evaluated as more favorable and acceptable by students in comparison with others interventions or control conditions. Considerations for future researches were discussed.

## Background

An important public health issue of our time is the excessive alcohol consumption and associated risk behaviors among college students. There is consistent evidence suggesting that young adults in college are drinking more than their non-college-attending peers and other populations [1,2]. College students in many countries are at elevated risk for heavy drinking, with serious short- or long-term health negative consequences. Several studies have reported a wide range of alcohol-related problems in college settings, including academic impairment, blackouts, violence, accidents, unprotected sexual behavior, other substance use, and alcohol dependence [3-5].

Preventive efforts during the formative college years may present an opportunity to change drinking behavior among students. Early detection and intervention are vital to reduce the number of alcohol-related problems in college campuses today [6]. Brief interventions (BIs) have emerged as a promising approach to provide early intervention, before or soon after the onset of negative consequences of alcohol consumption. There is convincing evidence for the efficacy and effectiveness of brief intervention in various healthcare settings [7,8].

In the academic context, several reviews have summarized the results of studies evaluating interventions to reduce heavy drinking among college students. For example, Cronce & Larimer [9] updated earlier qualitative reviews on individual-focused prevention and treatment approaches for college drinking [10,11]. The investigators supported the efficacy of skill-based and motivational interventions that incorporated personalized feedback, with or without an in-person intervention. More recently, Seigers & Carey [12] provided a critical review of the efficacy of brief interventions for alcohol use in college health centers and found similar results. BIs in these settings were considered acceptable and feasible for promoting risk reduction.

Carey et al. [13] conducted a meta-analysis evaluating 62 randomized clinical trials published between 1985 to early 2007. Results were similar to ours and support the efficacy of individual-focused alcohol interventions in reducing the quantity and frequency of alcohol use and alcohol-related problems among college students. However, this review considered an extensive variety of methodological conditions (i.e. interventions delivered via various modalities and heterogeneous conditions of sample). Our purpose was to evaluate specifically a standardized promising intervention named Brief Alcohol Screening Intervention for College Students (BASICS) [14], with methodological conditions more homogeneous that permit a more reliable comparison.

BASICS is a specific protocol of BI for college students delivered face-to-face and usually conducted over the course of two structured sessions, including motivational interview and personalized feedback based on student drinking behavior. It is especially relevant to encourage students to change their behavior by using empathy and warmth approach rather than confrontation. Moreover, clinicians can assist patients by helping them establish specific goals and build skills for modifying their drinking behavior.

Meta-analytic review often involves late-stage research and the resulting data should be of clinical and empirical value. A meta-analysis can clarify the current status of efficacy shown in the literature and help guide future research. Therefore, this article presents a systematic review and meta-analysis of the efficacy of BASICS in reducing alcohol consumption and associated problems among college students.

## Methods

### Criteria for considering studies for this review

Studies were considered for inclusion in this review according to predefined criteria as following:

#### Study design

Randomized controlled trials, especially designed to assess the efficacy of BI in reducing or preventing alcohol consumption and/or alcohol-related problems.

#### Subjects

College students engaged in heavy episodic drinking. Alcohol dependents and other substance users were excluded, considering recommendations and the target population of BASICS. Mandated or adjudicated college students were also excluded to maintain the homogeneity of the samples studied, and subjects with special motivational condition in relation to reducing them alcohol consumption and alcohol-related problems were not included either.

#### Interventions

Intervention was conducted according to the principles of BASICS protocol or very similar one. Studies have adopted three methodological conditions: (1) BI using techniques of motivational interview [15] and personalized feedback, (2) face-to-face intervention, and (3) comparison with other conditions (such as control group or alternative intervention).

#### Literature search

Relevant studies were identified by searching electronic bibliographic sources: Medline (1966 to 2011), PsycINFO (1840 to 2011), Web of Science (1898 to 2011) and Cochrane Library (December 2011). Search strategy used a combination of the following terms based on keywords and goals of this review:

*alcohol*: alcohol OR drinking OR binge [title/abstract]

*college students*: university OR universities OR college [title/abstract]

*efficacy*: treatment outcome OR efficacy OR effectiveness [title/abstract]

*BASICS*: brief intervention OR motivational OR prevention [title/abstract]

*randomized clinical trial*: clinical trial OR random*

These terms and combinations were adapted according to each database. No language restriction was applied. Additional studies were also searched by reading the reference list of the included articles and relevant systematic reviews.

**Figure 1:**
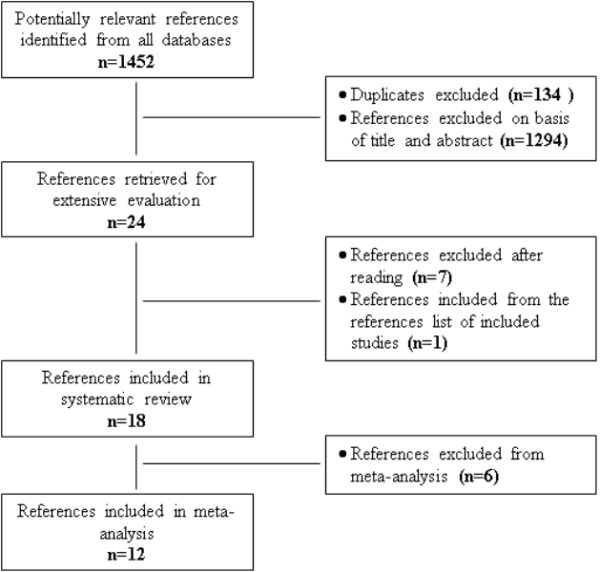
Sequence of the inclusion process of references in the systematic review and meta-analysis.

Following a search using the strategies and sources described, initial selection was based on information derived from title and abstract of all potentially relevant articles. Studies were reviewed for possible inclusion and full text was retrieved. All retrieved studies were assessed for inclusion in the review based on those criteria described above (*see* inclusion criteria for this review). Figure 1 provides a visual summary of the literature search, indicating the final sample for systematic review and meta-analysis.

### Data extraction

Identification and selection of potential studies and data extraction were conducted by the first author (Alexandre Fachini, AF). To ensure quality and accuracy, all these processes were independently reviewed by the second author (Poliana Patricio Aliane, PA). The third author (Edson Zangiacomi Martinez, EM) reviewed again the criteria when performing the statistics in meta-analysis. Any disagreement was resolved with discussion and consensus by the fourth author (Erikson Felipe Furtado, EF).

Data of each included study was tabulated and summarized according to the following variables: first author name, publication year, country, sample type, study design, interventions, type of data collection instruments (interviews, questionnaires, etc.), outcome measures, follow-up (retention and attrition), results, and conclusions. This information was submitted to critical analysis and considering qualitative and quantitative aspects.

### Assessment of methodological quality

An assessment of potential biases resulting from trial design was conducted independently by two of the authors. Quality assessment was based on the following aspects of methodology: selection bias (randomization sequence generation and allocation concealment), performance bias (blinding) and attrition bias.

#### Selection bias

It was analyzed if randomization occurs in an unpredictable sequence so that every participant had an equal chance of experiencing control or intervention conditions. Randomization sequence generation was classified as adequate (urn randomization, computer-generated, random number table, shuffled cards or tossing coins), unclear (not described) or inadequate (different approach from those considered adequate). Allocation concealment also was considered as adequate (central randomization, numbered or coded containers, and sequentially numbered opaque sealed envelopes), unclear (approaches not reported) or inadequate (alternate assignment and assignment by odd/even date of birth or day of the week).

#### Performance bias

Blinding of patients assignment or masking of clinicians regarding treatment condition is difficult to achieve in a trial evaluating a “talking therapy”, although this may be possible in cluster randomized trials; so, we noted the following: double blind, single blind (for outcome assessment in the follow-up) or unclear (condition not described, but supposedly interviewers were not blind).

#### Attrition bias

Differential loss of subjects from comparison groups was explored by recording how many participants were lost to follow-up in each group. Quality assessment considered if the loss to follow-up was completely recorded for each group and outcome measured, including presentation of significant differences between treatment and control groups regarding the loss to follow-up.

Moreover, a qualitative methodological score was assigned using a 12-item assessment of several aspects of methodological design [16]. Summary scores ranged from 0 to 17, with 14 or more points indicating excellent methodological quality.

### Meta-analysis

Two outcome measures were extracted for meta-analysis: alcohol consumption and alcohol-related problems. Alcohol consumption was defined as being the number of drinks per week and alcohol-related problems as the mean score obtained with specific instrument used to assess this variable (i.e. Rutgers Alcohol Problem Index, RAPI).

The difference between the mean values regarding outcomes of interest (alcohol consumption and alcohol-related problems), comparing intervention and control groups, was used as measure of the treatment effect. Heterogeneity across the studies was assessed by using the Cochran’s Q test. When the p-value for heterogeneity in any analysis was less than 0.10, the random effects meta-analysis model was used for calculating the summary measures. The meta-analysis was based on combining estimates of a treatment difference across trials, assuming that the treatment difference parameters in the studies are a sample of independent observations from a normal distribution and introducing normally distributed random effects taking into account the heterogeneity across the studies [17]. The random effects model estimates summary difference between the groups with their respective 95% confidence intervals (95% CI). When this confidence interval did not include zero, then the model concluded that there was a significant difference between the groups. R software was used for this statistical analysis.

## Results

The search strategy found 1452 potentially relevant published studies by screening the web (Figure 1). Of these, 134 references were found to be duplicated, 1294 references were excluded based on their titles and abstracts and, finally, 24 selected articles were retrieved for detailed evaluation of the full published version. Of these, we excluded seven articles as they did not match the criteria and inserted one that was found by searching and reading carefully the list of references of the included articles. The final sample of studies in the systematic review had 18 randomized clinical trials. Table 1 describes the key characteristics of the included studies such as author, year, sample, follow-up, retention, methodology, risk of bias, and methodological quality score (MQS).

**Table 1:** Summarized description of studies included in the systematic review

**Author (year)**	**Sample**	**Follow-up**	**Methodology**	**MQS**^**a**^
	**N (%male); mean age**	**(% retention)**		
Wagener et al. [18]	152 (54.6%); 20.9 years	10 months (94.7%)	randomization sequence: *adequate*	11
			allocation concealment: *unclear*	
			blinding: *unclear*	
Fernandez et al. [19]	1014 (43%); 18.4 years	10 months (90.8%)	randomization sequence: *adequate*	13
		22 months (84%)	allocation concealment: *unclear*	
		12 months for parents	blinding: *blind* (for interviewers)	
Mastroleo et al. [20]	122 (52%); 18.1 years	3 months (85.4%)	randomization sequence: *adequate*	12
			allocation concealment: *unclear*	
			blinding: *unclear*	
Murphy et al. [21]	study 1: 74 (41%); 21.2 years	1 month	randomization sequence: *adequate*	11
	study 2: 133 (50%); 18.6 years	(study 1: 93.2%)	allocation concealment: *unclear*	
		(study 2: 88.7%)	blinding: *blind* (for interviewer)	
Turrisi et al. [22]	1275 (44.4%); 17.9 years	10 months (85.5%) 12 months for parents	randomization sequence: *adequate*	14
			allocation concealment: *unclear*	
			blinding: *unclear*	
Schaus et al. [23]	363 (48%); 20.6 years	3 months (76%)	randomization sequence: *adequate*	13
		6 months (58%)	allocation concealment: *adequate*	
		9 months (59%)	blinding: *unclear*	
		12 months (65%)		
Butler et al. [24]	84 (34.3%); 20.2 years	1 month (73.6%)	randomization sequence: *unclear*	11
			allocation concealment: *unclear*	
			blinding: *unclear*	
Simão et al. [25]	266 (56.4%); 19.6 years	12 months (98.8%)	randomization sequence: *unclear*	12
		24 months (81.2%)	allocation concealment: *unclear*	
			blinding: *unclear*	
Wood et al. [26]	335 (47.5%); 20.9 years	1 month (82.4%)	randomization sequence: *unclear*	12
		3 months (75.5%)		
		6 months (72.5%)	allocation concealment: *unclear*	
			blinding: *unclear*	
Carey et al. [27]	509 (35%); 19.2 years	1 month (97%)	randomization sequence: *unclear*	14
		6 months (87%)	allocation concealment: *unclear*	
		12 months (78%)	blinding: *unclear*	
Murphy et al. [28]	54 (31%); 19.9 years	6 months (94%)	randomization sequence: *unclear*	12
			allocation concealment: *unclear*	
			blinding: *unclear*	
Larimer et al. [30]	159 (not described);18.8 years	12 months (75%)	randomization sequence: *unclear*	13
			allocation concealment: *unclear*	
			blinding: *unclear*	
Baer et al. [31]	659 (45%); ≤ 19 years	12 months (not reported)	randomization sequence: *unclear*	13
		6 months (>72%)	allocation concealment: *unclear*	
		12 months (>72%)	blinding: *unclear*	
		24 months (93.5%)		
Roberts et al. [32]	390 (44.8%); not described	24 months (84.2%)	randomization sequence: *unclear*	14
			allocation concealment: *unclear*	
			blinding: *unclear*	
Borsari et al. [33]	63 (43%); 18.6 years	6 week (98%)	randomization sequence: *adequate*	11
			allocation concealment: *unclear*	
			blinding: *unclear*	
Marlatt et al. [34]	348 (45.9%); not described	6 months (100%)	randomization sequence: *adequate*	15
		12 months (94%)	allocation concealment: *unclear*	
		24 months (88%)	blinding: *unclear*	
Baer et al. [35]	134 (48%); 21.2 years	3 months (>72%)	randomization sequence: *unclear*	11
		6 months (>72%)	allocation concealment: *unclear*	
		12 months (>72%)		
		24 months (93.5%)		

^a^ MQS, Methodological Quality Score.

### Participants and recruitment

A total of 6233 college students participated in the studies with sample sizes ranging from 54 to 1275 (Median = 212) and the proportion of women was slightly higher than that of men in most of the studies. Participants had a mean age of 20 years approximately. All participants were from public universities and identified as at-risk drinkers. Studies used screening tools for alcohol consumption and alcohol-related problems such as AUDIT, DDQ, MAST, YAAPST, and RAPI. Most of the studies used a combination of these tools and determined alternative inclusion criteria to fit them, thus increasing the likelihood of picking up relevant participants. Screening tools were applied by means of telephone, computer, mail and face-to-face interview. Inclusion criteria in terms of at-risk drinking were defined by the screening total score, level of binge drinking or number of drink units per week.

### Description of the groups

#### Brief intervention group

Trials evaluated one (72%) or two (28%) brief intervention sessions. The duration of individual sessions varied from 30 to 90 minutes and only one trial used an extended intervention with a 30-minute booster session [14]. Professionals administering the intervention were usually psychologists and advanced peers, but psychiatrist and social worker participated in one trial [20]. Professionals were trained and supervised in most of the studies (84%).

#### Control group

Three categories of control were used. Most trials administered no intervention or made only an evaluation, followed by usual treatment (i.e. advice to cut down drinking) and other alternative interventions (computerized programs, Alcohol Expectancy Challenge, parent and educational interventions). Treatment duration in the control condition ranged from 11 to 90 minutes. Some trials used more than one control conditions.

### Risk of bias

#### Randomization sequence

Randomization was found to be adequate in eight trials. The methods of randomization used were computer-generated sequence (four trials), flip of a coin (two trials), urn and random number table (one trial each). In the remaining 10 trials the method of randomization sequence was unclear. No trial described an inadequate randomization sequence.

#### Allocation concealment

Only one trial described an adequate process of allocation concealment by using sealed envelopes [18]. In the other 17 trials, allocation concealment was considered unclear and none was found to be inadequate.

#### Blinding

Due to the nature of the interventions used, it was not possible to blind either participants or providers of care. In two trials the interviewers were blinded to the outcomes. This was not reported in any other trial and no inadequate blinding was described.

### Duration of the trials (follow-up)

Post-treatment assessments were conducted over a period of time ranging from 1 to 48 months, with 11 different follow-up periods. Nine studies (50%) conducted assessments at multiple time points. The most common period of follow-up was that lasting 12 months (seven trials), followed by those lasting six (six trials), three and 24 months (five trials).

Retention rates in the last follow-up ranged from 65% to 98%. Studies reported no significant differences between participants who completed the post-treatment assessment and those who did not.

### Methodological quality score

All studies were assessed according to the methodological quality score. Scoring ranged from 11 to 15, indicating a good methodological quality. Of the 18 studies reviewed, four trials were considered to have an excellent methodology [17,22,27,29]. Common methodological problems were the lack of collateral or objective verification, non-blind follow-ups, lack of parallel replication by separate research teams, and short follow-ups.

### Effect of intervention

**Figure 2:**
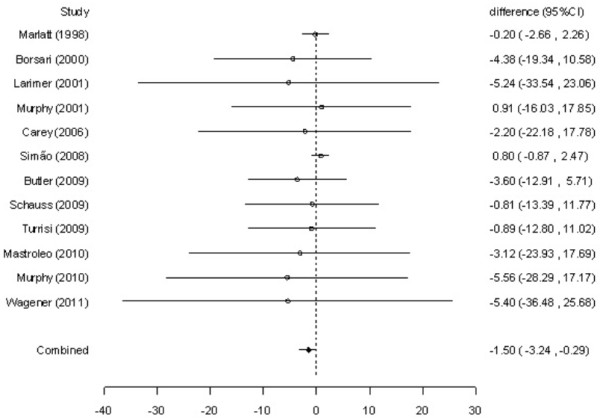
Meta-analysis of twelve randomized clinical trials about data on alcohol consumption (Q-statistics = 32.61, 11 df, p < .01).

**Figure 3:**
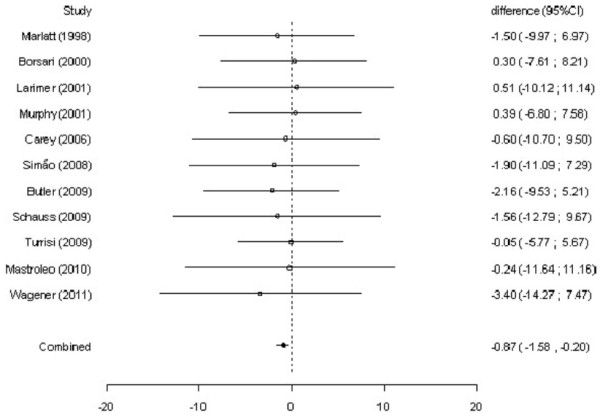
Meta-analysis of eleven randomized clinical trials about data on alcohol-related problems (Q-statistics = 21.38, 10 df, p = .02).

The simple differences at approximately 12 months of follow-up appeared to be heterogeneous for mean reductions in alcohol consumption (Q-statistics = 32.61, 11 degrees of freedom [df], p < .01) and alcohol-related problems (Q-statistics = 21.38, 10 df, p = .02). Thus, a random-effect model was used in order to estimate a combined measure of the effect of intervention. At approximately 12 months, students receiving BASICS had a significant reduction in alcohol consumption (difference between means = −1.50 drinks per week, 95% CI: -3.24 to −0.29) and alcohol-related problems (difference between means = −0.87, 95% CI: -1.58 to −0.20) compared to controls. Figures 2 and 3 show the mean reductions in alcohol consumption and alcohol-related problems, respectively, at a 95% CI in each study included in the meta-analysis. In addition, it is also shown the combined measure of the effect of intervention obtained by the random-effect models and their respective 95% confidence intervals. Considering that these confidence intervals do not include zero, there are significant differences between the groups regarding the mean reductions in alcohol consumption and alcohol-related problems.

## Discussion

Meta-analysis of randomized controlled trials combines information from independent studies that address a similar question to provide more reliable estimates of treatment effects, thus providing valuable information for researchers, policy-makers, and clinicians. Firstly, our meta-analysis may be useful for summarizing the available information and generating hypotheses for future researches. Secondly, and equally important, we have found a great effect size, which indicates the efficacy of BASICS. The results of the meta-analysis procedure support that brief intervention seems to be more efficacious in reducing both heavy drinking and alcohol-related problems among at-risk college students in comparison with control groups after 12 months of follow-up. A counselor-administered motivational interview (MI) plus feedback may be an ideal first-line intervention for heavy drinking college students. Review indicates some other important aspects to consider about mechanisms and characteristics of the BASICS method.

### Moderators of the intervention

Results of the studies pointed to some possible moderators for the effects of intervention. One of these moderators was gender. Some authors have not found a significant interaction between gender, thus indicating that BASICS resulted in comparable drinking reductions in both women and men [22,26,34]. Nevertheless, Marlatt et al. [34] observed that women showed significantly greater decrements in drinking problems over time than men. On the other hand, Butler & Correia [25] and Murphy [21,28] reported that BASICS was more effective in women, indicating slightly greater reductions in drinking compared to men. As argued by Borsari & Carey [36], women may be less reliant on drinking situations and social drinking to meet their social needs.

A second possible moderator was the age of the students. Baer et al. [35] observed that treatment response was related to age, as the subjects showed increased drinking behavior during the year they reached legal drinking status. Marlatt et al. [34] claim that it is important to note that high-risk students, both in intervention and control groups, showed a significant drop in drinking rates and related problems over time, suggesting an effect of the maturational process. Some years later, Baer et al. [31] observed that brief intervention for high-risk college drinkers can achieve long-term benefits even in the context of maturational trends. However, it remains unclear as to how this maturational acceleration itself is mediated (by enhanced motivation, heightened personal awareness of risk, improved drinking habits and coping skills, or some combination thereof). According to Schulenberg et al. [37], further research is needed to differentiate between those heavy drinkers in college who go on to future alcohol abuse and dependence and those whose drinking habit decreases after college.

Finally, the perceived alcohol peer norms mediated the effects of intervention in comparison with the control group for all drinking outcomes. Results reported by Larimer et al. [30] and Turrisi et al. [22] suggest that a peer-delivered feedback intervention may be an important aspect of this preventive strategy, especially with fraternity members [30]. These findings suggest that trained peer counselors are as effective as professionals in encouraging drinking changes among college students, a finding also discussed elsewhere [38,39]. However, a disadvantage is that peer providers require considerable training and supervision. Most research protocols recommend weekly individual or group supervision by a trained therapist. Mastroleo et al. [20], for example, observed that post-training supervision enhance micro-skills during a BASICS intervention for peer counselors and improve BASICS fidelity.

### Comparison with other interventions

Participants were given a more favorable rating for BASICS than other interventions or control conditions in several studies, besides presenting a higher reduction of alcohol consumption [18,21,24,33,34]. In the study conducted by Marlatt et al. [34], participants indicated that they would recommend the interview to a friend. In addition, they characterized the interviewer as well-organized, competent, well-trained, warm, and understanding. In the Borsari and Carey’s [33] study, the participants reported high levels of satisfaction with the intervention.

Murphy et al. [21] suggest that students may find the counselor-administered MI interventions, such as the BASICS, more interesting, credible, and useful than other ones (e.g. computerized interventions). This preference information may be relevant to the universities’ efforts to encourage and increase the participation in alcohol interventions. Moreover, the change in drinking of college students provides qualified support for the superiority of BASICS over other interventions. It is also possible that the advantage of BASICS on this respect is related to the fact that in-person interventions elicit a greater commitment or social desirability from the participants, which might in turn lead to subjective appraisals of change that overestimate actual changes in drinking behavior [40,41].

Turrisi et al. [22] and Fernandez et al. [19] pointed to another important issue. They reported that the parent intervention delivered to students before they begin college serves to enhance the efficacy of BASICS intervention, potentially priming the students to respond to subsequent BASICS sessions. Although more research is necessary about this topic, these data indicate the relevance of including parents in preventive actions to change the drinking behavior of the students in the college.

## Conclusions

The results of the meta-analysis bring reliable evidence supporting the efficacy of the BASICS method in reducing alcohol consumption and its negative consequences among college students.

Researchers should consider the use of some methodological quality criteria such as the inclusion of collateral data or other validation procedure of self-reported drinking, as well as the planning of longer follow-ups and a blind interviewer at post-assessment intervention. Moreover, in order to intensify the results, efforts can be made to capitalize on the substantial decreases in drinking, which are evident shortly after the intervention. For example, this could be achieved by using booster sessions to maintain initial decreases in consumption.

Another special issue about BASICS is the influence of mechanisms of change. Identifying and isolating the mechanisms of change that lead to a reduction in the risk for heavy drinking among college students – a high-risk population, will allow for the development of succinct, targeted, and thereby more effective interventions. For example, it appears that there may be some utility in using gender-specific interventions and also possibly in including peer providers in the process. Similarly, the association with a parent intervention may maximize the BASICS intervention.

In sum, BASICS can help heavy college drinkers to reduce or stop drinking and screen alcohol-dependent students by motivating them to enter treatment. Besides, face-to-face interview, a characteristic of BASICS, appears to improve the overall perception about healthier habits among the college students. The reduction in drinking levels and alcohol- related problems among high-risk college students can result in a corresponding decrease in medical and societal costs. BASICS presents other benefits to be considered for health policy-makers as it can be delivered by any trained assistant at a low-cost implementation.

The results of this systematic review using meta-analysis shows the relevance of further clinical research aimed at identifying potential early predictors of change in drinking behaviors. It could help not only to improve the current content of BASICS but also create an opportunity to introduce new components to treatment. Finally, from a public health perspective, the evidence of a successfully reliable prevention method for reduction of risk behaviors may benefit students themselves, their lives and professional future as well the academic community.

## Competing interests

The authors declare that they have no competing interests.

## Authors’ contributions

AF participated of all process of preparation and development of systematic review, meta-analysis, and manuscript. PAP reviewed the literature search, inclusion of articles in the systematic review, and quality assessment of the studies. EZM reviewed inclusion of articles in meta-analysis, conducted the statistical analyses and wrote that section. EFF developed eligibility criteria, judged disagreements on articles included in the systematic review, and reviewed the manuscript. All authors contributed to and have approved the final manuscript.
